# MMI - An unbiased approach to health education selection?

**DOI:** 10.15694/mep.2018.0000111.1

**Published:** 2018-05-28

**Authors:** Gerens Curnow

**Affiliations:** 1 University of Exeter Medical School

**Keywords:** Multiple Mini Interview, Bias

## Abstract

This article was migrated. The article was marked as recommended.

Background

Widening participation to health science degrees is a vital part of our efforts to ensure a high-quality pool of graduates in clinical fields. With the increasing usage of multiple mini interviews for selection to these courses, it is crucial that we recognise the biases within these selection processes.

Aims

This paper aims to examine recently published literature to determine the extent to which demographic factors (age, race, sex and socio-economic background) impact on MMI scores for applicants to health science degrees.

Methods

A literature search was conducted using the Medline and SCOPUS databases for literature published from 2015 to present. Relevant papers were identified through a Boolean search, and individually analysed to determine their relevance to this review.

Results

This review identified nine relevant papers. Biases were identified in all four domains, but the evidence was mixed and of varying quality. The strongest evidence for a bias was found in papers looking at socio-economic background.

Conclusion

Further research is required to determine the extent to which the MMI approach is biased against certain groups of applicants, and to identify ways to address these imbalances, as evidence of the impact of demographics on MMI score has been identified.

## Introduction

The topic of Widening Participation (WP) is experiencing a lot of attention at present. A recent consultation by the Department of Health on expanding undergraduate medical school places includes a focus on how universities will ensure they are recruiting underrepresented demographics, which highlights the current atmosphere with regards to WP ( Department of Health 2017). Given the relatively low attrition rates in many health science degrees ( Rees et al. 2016), ensuring that the selection process for entry onto these courses is reliable and valid is of paramount importance.

A broad range of attributes and skills, both cognitive and non-cognitive, is vital for everyone working in the clinical environment (Ferguson et al. 2002). Deciding which attributes to test poses a challenge for health science institutions. Of all the tools available to selection committees, from standardised admission tests, such as the UK Clinical Aptitude Test or the Pharmacy College Admissions Test, to personal statements and academic achievement, the interview has perhaps undergone the most change in recent years.

Interviews allow institutions to assess candidates against an extremely wide range of criteria, depending on the assessment aims of each institution; from biomedical knowledge and statistical ability, to communication skills and empathy. It is perhaps surprising therefore that there are only two main interview modalities that are currently employed by health science institutions: Multiple Mini Interviews (MMI’s) and panel interviews ( Rees et al. 2016).

MMI’s were first developed in 2004 to improve upon the relatively poor predictive validity of the contemporary non-cognitive assessments, such as panel interviews and personal statements ( Rees et al. 2016; Reiter et al. 2007). Their uptake by health science degree providers has been rapid, and much research has been carried out into their efficacy as a selection tool.

Given the current emphasis on WP, and the importance of the selection process to health science degrees in determining the make-up of the workforce in health professions, it is vital that any hidden biases within the MMI system are well understood. Bias in health science selection procedures, which refers to the phenomenon wherein attributes other than those designed to be assessed (e.g. race, age, socio-economic background) affect performance in an assessment ( Rees et al. 2016), has the potential reduce the quality of the workforce by eliminating potentially high-performing candidates.

This review will examine the recently published literature to investigate the extent to which MMI’s can be discriminatory in terms of age, sex, socio-economic background (SEB), and race. This will update the findings of the BEME Guide No. 37, which provided an insight into the literature on this topic from 2004-2014 ( Rees et al. 2016). While differences in the success rates of these candidates may not necessarily indicate a bias within the system (e.g. if desirable traits tend to exist more commonly in one group than another), it will provide a useful metric for assessing the validity of the assessment and provide a grounding for further research on this topic.

## Search Methods

The literature search for this review was conducted on Medline and SCOPUS on 15 ^th^ October 2017. The search used to generate the literature for this review was:

(mmi OR “multiple mini interview*” OR “multiple-mini-interview*” OR “multiple-mini interview*”) AND (sex OR socioeconomic OR socio-economic OR bias OR race OR aboriginal OR age)

**Figure 1.**
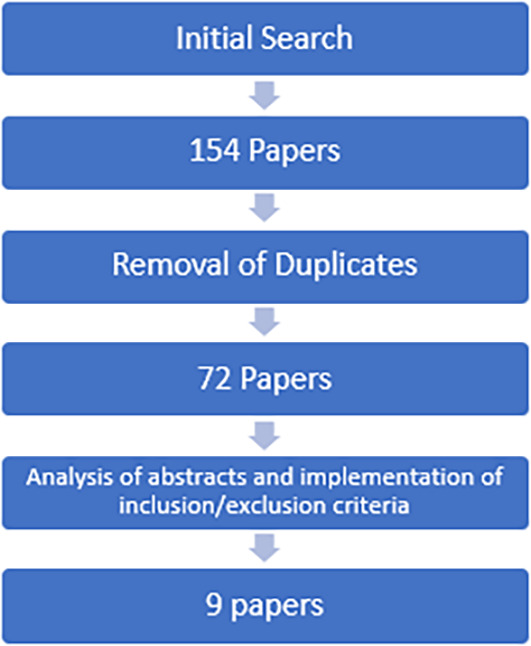
Process for Selecting Papers

The search was limited to papers published since 2015 to ensure it provided an update to the BEME Guide No. 37, the literature search for which was updated in 2014 ( Rees et al. 2016). This is a narrower search than the one used for the BEME review, as the present study is looking only at bias and not at face validity, acceptability etc. This search revealed 154 papers, which were assessed against inclusion and exclusion criteria ( table one), leaving nine papers for use in the review (see Figure 1).

**Table One.** Inclusion and Exclusion Criteria

Inclusion Criteria	Exclusion Criteria
• Primary research • Examining bias within MMI interviews in a health sciences setting • At least one of the following examined in terms of bias: • Race • Age • Sex • Socioeconomic Background • Published since 2015	• Not primary research • Not health sciences specific • Published before 2015 • Paper not in English

## Results

The nine papers identified examined entry onto three different health science courses: six for medicine, two for nursing, and one for pharmacy. A summary of these papers can be found in table 2.

**Table 2.**
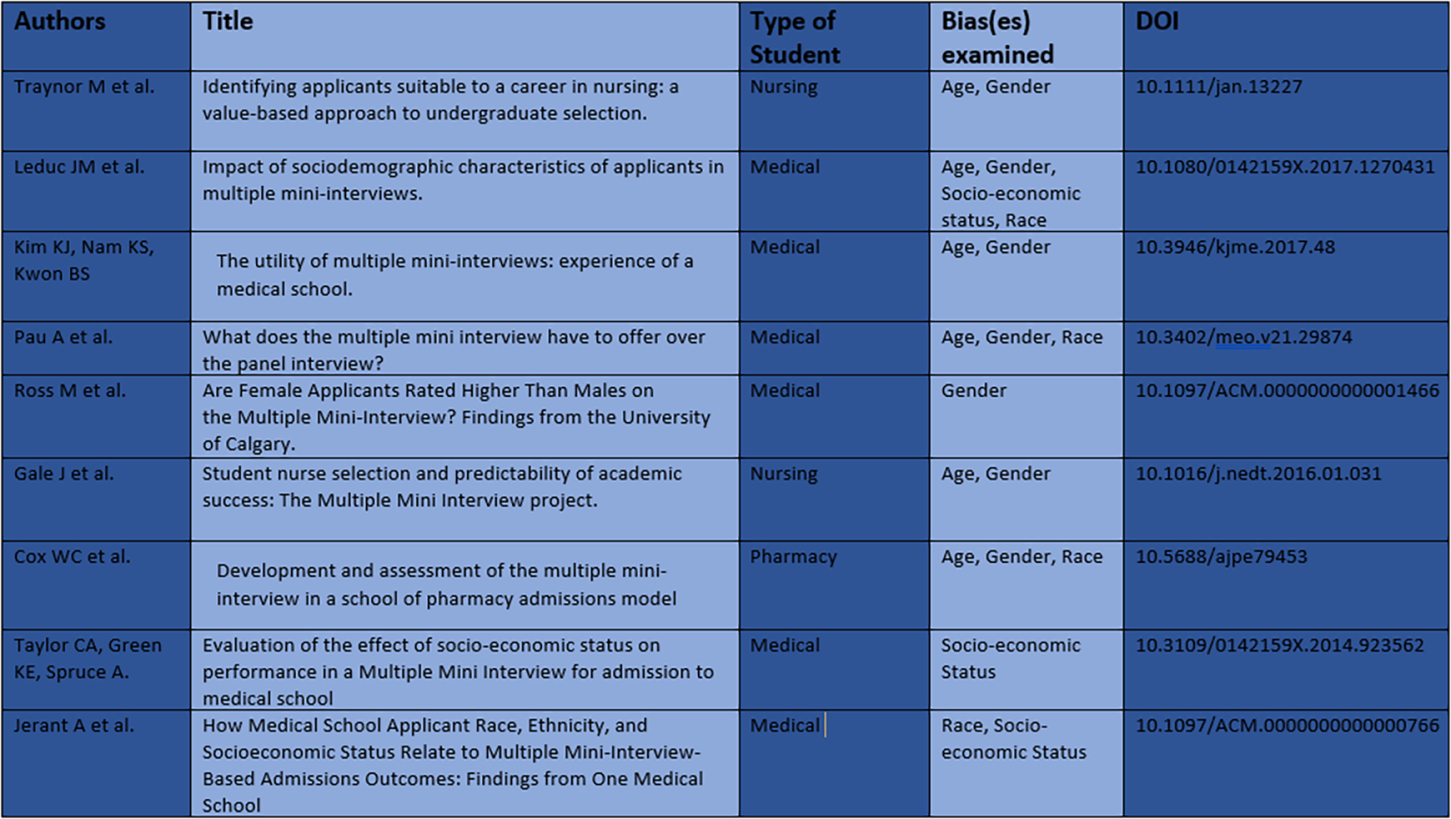
Summary of Papers

## Discussion

### Age

Six papers investigated the impact of applicants’ age on MMI score; three in applicants to medicine, two to nursing, and one to pharmacy ( Traynor et al. 2017; Leduc et al. 2017; Kim et al. 2017; Pau et al. 2016; Gale et al. 2016; Cox et al. 2015). Two studies found age did impact on MMI score, while four found it didn’t.

Traynor et al. ( Traynor et al. 2017) found that being older provided a small but significant increase in MMI score in nursing students at Queen’s University Belfast (n=110). They reported that, for every 10 extra years of age, candidates’ MMI scores improved by 5 points (an increase of 0.5 SD, p=0.008). This study was conducted on 1 ^st^ year students, who had already passed a panel-based interview and gained entry to the course, which may have impacted the results. It was also heavily dominated by female students (92%), which limits the generalisability of the findings.

Leduc et al. ( Leduc et al. 2017) used the self-reported socio-economic and demographic data from 1,089 applicants to three French-speaking Canadian medical schools, to examine the extent to which these factors impacted MMI scores. Their analysis revealed that candidates aged 25-29 had a small but significant advantage over those aged <20 years (p=0.001). While this study has the advantage of a large sample size, all MMI’s were conducted in a single centre. Further, it is unclear what impact the fact that the interviews were conducted in French might have on the study’s generalisability.

Four other studies (total n=754) found no impact of age on MMI result ( Kim et al. 2017; Pau et al. 2016; Gale et al. 2016; Cox et al. 2015). These findings mirror the BEME Guide No. 37, which also reported conflicting results regarding the impact of age on MMI score ( Rees et al. 2016). These conflicting results, coupled with the lack of adjustment for confounding factors in these studies, suggests that this is an area that requires further research.

### Sex

Seven papers investigated the effect of sex on MMI score. Four papers examined medical school admissions; two, nursing school admissions; and one, pharmacy admissions. Two papers identified a significant impact of sex on MMI score, while the other four did not.

Leduc et al (2017) found that female students performed significantly better in MMI’s for three French-Canadian medical schools than their male counterparts, but only in the subgroup that spoke French at home (p=0.003). This implies a previously unexplored interaction between sex and language, which warrants further study.

Ross et al (2017) examined the MMI scores of 526 medical applicants at the University of Calgary. It found that female students achieved a higher score than their male counterparts (p<0.001), even when they controlled for confounding variables (e.g. MCAT score, applicant age etc). Two interpretations for this difference are proposed. Firstly, that female applicants are more likely to possess the attributes of interest in the MMI assessment (communication skills, critical thinking, ethical decision making etc) which, if true, this would support the validity of the MMI process. The other is that the interviewers are victims of confirmation bias, in that they *expect* female applicants to possess these skills, and therefore mark them more highly. The authors of this study recommend a longitudinal, health outcome based approach to investigate this further. None of the other studies included in this analysis showed any significant effect of sex on MMI scores ( Traynor et al. 2017; Kim et al. 2017; Pau et al. 2013; Gale et al. 2016; Cox et al. 2015).

### Race

Four studies investigated the impact of race on MMI scores; three on medical school applications, and one on pharmacy ( Leduc et al. 2017; Pau et al. 2016; Cox et al. 2015; Jerant et al. 2015). One of these studies reported a significant impact. Leduc et al ( Leduc et al. 2017) found that applicants who self-declared as “Chinese” or “Southeast Asian” performed significantly worse than all other ethnic groups (p=0.013, 0.003 respectively). It also found that applicants who identified as “White/Caucasian” performed significantly better than other ethnic groups (p=0.012). In a qualitative investigation of the potential reasons behind this, it was reported that many of the interviewers had come across a stereotype that Asian students are “shy”, and that this may have impacted on the scores given through a confirmation bias. The other three papers reported no significant impact.

### Socio-economic Status

Three papers analysed the impact of socio-economic background (SEB) on MMI scores ( Leduc et al. 2017; Taylor et al. 2015; Jerant et al. 2015).

Leduc et al. used a self-assessment form to categorise applicants into those with a family income of either <$100,000, $100,000 to $250,000, or >$250,000, and compared the MMI scores for applicants within these categories. It found that students who declared a parental income of >C$250,000 had significantly higher MMI scores (M=252.8) than those in the C$100,000 to C$250,000 (M=243.3, p=0.035) and <C$100,000 (M=237.1, p<0.001) groups. This study is limited by the fact that it relies on the applicants’ self-assessment of family income, but nonetheless draws into question the validity of the process.

Jerant et al. ( Jerant et al. 2015) examined eight different factors, including parents’ education level, whether the applicant had contributed to family income, and whether the applicant was in receipt of income support, to create a composite score for SEB. This was applied to 1,420 MMI interviewees at the University of California. They found that candidates from a more deprived background had a significantly lower MMI score (p=0.03), but that this was only a small a small impact of less than 0.2 S.D. However, being from a more disadvantaged background was associated with a greater probability of being accepted onto the course (OR 3.28, p>0.001), since the final decision was made on a wide range of factors beyond MMI score, including personal statements and MCAT scores. This shows that biases within the MMI system can be compensated for by the admissions process as a whole.

Taylor et al. ( Taylor et al. 2015) found no significant relationship between the MMI score achieved by an applicant and the status of the applicant’s school (selective/fee paying vs non-selective and non-fee paying). Nor was there a relationship between MMI score and Higher Education participation rates in the area in which the applicant lived.

## Study Limitations

This study has several limitations. As discussed previously, it does not examine the reasons behind differences in success rates among applicants with different demographics. While confirmation bias in the examiners may explain these differences, in many instances the true reasons could be more complex, with an interplay of valid and invalid factors influencing the score candidates receive. Further, this review has not examined the impact of the demographics of the assessor on the levels of bias within the MMI system, which presents an interesting topic for further study. Lastly, this review has presented no evidence regarding the ways in which these impacts should be mitigated.

## Conclusion

This review has found mixed evidence for the impact of demographics on MMI score for the selection of health science students. For all aspects examined in this review (age, sex, socio-economic background, and race), there is some evidence of bias within the MMI process, most convincingly in terms of candidates’ socio-economic backgrounds. This demands further research, with the aim of determining the long-term impact of bias on health outcomes, and the methods through which these biases can be mitigated.

## Take Home Messages

• MMI’s have been increasingly employed in recent decades
• The last major investigation into the validity of this assessment type was completed in 2014
• This review has found mixed evidence, with some studies indicating the presence of bias
• Educators should take steps to mitigate the impact of these biases
• More research is needed to investigate the potential reasons behind this data

## Notes On Contributors

Gerens Curnow is an undergraduate medical student at the University of Exeter, currently undertaking an MSc in Clinical Education.
