# Supplementary material for: MMI - An unbiased approach to health education selection?
Source: MedEdPublish (2016). 2018 May 28;7:111. [Version 1] doi: 10.15694/mep.2018.0000111.1 (PMC10699372; doi:10.15694/mep.2018.0000111.1)
Supplement: Peer review history

# Reviewer report: Reviewer response for version 1

**DOI:** 10.21956/mep.19584.r29069

**Published:** 2019-02-23

**Authors:** Trevor Gibbs

**Affiliations:** 1 AMEE

This review has been migrated. The reviewer awarded 4 stars out of 5 A very well written and balanced paper with some new references on the subject. Although there are quite a few papers now in circulation regarding the MMIs, there seems to be work still to be done, especially in the area of how MMIs probably need to be adapted to the widening access admission programmes.A paper all involved in student selection should read

Reviewer Expertise:

NA

No decision status is available

# Reviewer report: Reviewer response for version 1

**DOI:** 10.21956/mep.19584.r29071

**Published:** 2018-05-31

**Authors:** David Bruce

**Affiliations:** 1 None

This review has been migrated. The reviewer awarded 4 stars out of 5 This is a well written and interesting paper which covers an important topic. The MMI is increasingly used in student selection, and this paper which covers research from 2014 onwards adds to the evidence produced in the BEME guide. Although the results show a mixed picture in how the age, gender, race and socio-economic status of applicants can effect success at MMI, some evidence of decreased performance in those from lower socio-economic groups is an important finding in the drive to widen access to the health professions. This calls for more research and a balance of admissions criteria. An enjoyable read that will be of great interest to all involved in student admissions.

Reviewer Expertise:

NA

No decision status is available

# Reviewer report: Reviewer response for version 1

**DOI:** 10.21956/mep.19584.r29068

**Published:** 2018-05-29

**Authors:** Sateesh Babu Arja

**Affiliations:** 1 Avalon University School of Medicine

This review has been migrated. The reviewer awarded 4 stars out of 5 Very thoughtful paper and I enjoyed reading it. The four demographics selected that were selected for this study are Age, sex, race, and socioeconomic background are very interesting. Mni-multiple interviews itself is a very curious topic in the current medical students' selection and admission process. There are many articles in favor of MMI vs Traditional interviews. But the same way we have some papers in the literature offering no special added advantage with MMI. One such paper published recently in Academic Medicine Journal is "Do Multiple Mini-Interview and Traditional Interview Scores Differ in Their Associations With Acceptance Offers Within and Across Five California Medical Schools?"Thank you for providing the detailed analysis based on the literature regarding the bias associated with age, sex, race, and socio-economic background for scoring in MMI. There was no conclusive evidence as author quoted. It requires further research. This paper is interesting for interviewers and admissions committees of the medical programs.

Reviewer Expertise:

NA

No decision status is available

# Reviewer report: Reviewer response for version 1

**DOI:** 10.21956/mep.19584.r29070

**Published:** 2018-05-28

**Authors:** P Ravi Shankar

**Affiliations:** 1 American International Medical University

This review has been migrated. The reviewer awarded 4 stars out of 5 This is an interesting study examining the impact of four demographic characteristics (age, gender, race and socioeconomic background) on performance in multiple mini-interviews (MMIs). The methodology of the study/review is well described. Limiting the search to the Medline and SCOPUS databases may have excluded some studies dealing with this topic. MMI is being increasingly used for admission to health profession schools. The paper is well written. Educators involved in admissions to health professions schools will find this manuscript of special interest.

Reviewer Expertise:

NA

No decision status is available
